# Adenosinergic System Involvement in Ischemic Stroke Patients’ Lymphocytes

**DOI:** 10.3390/cells9051072

**Published:** 2020-04-25

**Authors:** Silvia Pasquini, Fabrizio Vincenzi, Ilaria Casetta, Michele Laudisi, Stefania Merighi, Stefania Gessi, Pier Andrea Borea, Katia Varani

**Affiliations:** 1 Department of Morphology, Surgery and Experimental Medicine, Pharmacology Section, University of Ferrara, 44121 Ferrara, Italy; psqslv@unife.it (S.P.); mhs@unife.it (S.M.); gss@unife.it (S.G.); vrk@unife.it (K.V.); 2 Department of Biomedical and Specialist Surgical Sciences, Section of Neurological, Psychiatric and Psychological Sciences, University of Ferrara, 44121 Ferrara, Italy; ilaria.casetta@unife.it (I.C.); michele.laudisi@unife.it (M.L.); 3 University of Ferrara, 44121 Ferrara, Italy; bpa@unife.it

**Keywords:** adenosine, adenosine receptors, A_2A_ adenosine receptors, ischemic stroke, lymphocytes, CD39, CD73

## Abstract

Adenosine modulates many physiological processes through the interaction with adenosine receptors (ARs) named as A_1_, A_2A_, A_2B,_ and A_3_ARs. During ischemic stroke, adenosine mediates neuroprotective and anti-inflammatory effects through ARs activation. One of the dominant pathways generating extracellular adenosine involves the dephosphorylation of ATP by ecto-nucleotidases CD39 and CD73, which efficiently hydrolyze extracellular ATP to adenosine. The aim of the study is to assess the presence of ARs in lymphocytes from ischemic stroke patients compared to healthy subjects and to analyze changes in CD39 and CD73 expression in CD4^+^ and CD8^+^ lymphocytes. Saturation binding experiments revealed that A_2A_ARs affinity and density were significantly increased in ischemic stroke patients whilst no differences were found in A_1_, A_2B,_ and A_3_ARs. These results were also confirmed in reverse transcription (RT)-polymerase chain reaction (PCR) assays where A_2A_AR mRNA levels of ischemic stroke patients were higher than in control subjects. In flow cytometry experiments, the percentage of CD73^+^ cells was significantly decreased in lymphocytes and in T-lymphocyte subclasses CD4^+^ and CD8^+^ obtained from ischemic stroke patients in comparison with healthy individuals. These data corroborate the importance of the adenosinergic system in ischemic stroke and could open the way to more targeted therapeutic approaches and biomarker development for ischemic stroke.

## 1. Introduction

Adenosine is one of the most important nucleosides in the human body, derived from the backbone of adenosine triphosphate (ATP) and strictly implicated in the regulation of a large number of physiological and pathological signals [1,2]. Adenosine is able to exert its functions through the interaction with four adenosine receptors (ARs), belonging to the family of transmembrane G protein-coupled receptors (GPCRs) named as A_1_, A_2A_, A_2B_, and A_3_ [3,4]. 

Ischemic stroke is a complex pathology characterized by the sudden loss of blood circulation to an area of the brain and results in a corresponding loss of neurologic function [5]. The primary damage following an ischemic insult is due to the massive increase of extracellular glutamate, subsequently occurs the activation of resident immune cells, such as microglia, and production or activation of inflammation mediators [6]. The extracellular concentration of adenosine increases during ischemia with values in the range of 1000 nM in the first 20 min and return to basal values after about 4 hours [7]. The principal mechanism involved is represented by the hydrolysis to adenosine of extracellularly released ATP by ecto-nucleotidases named as CD39 and CD73 [8]. Adenosine seems to have a role as an endogenous mediator of neuroprotection in the homeostatic response to changes occurring during ischemia [9]. As a consequence, ARs could be interesting targets for therapeutic implementation in the treatment of stroke given the rise in adenosine concentration after ischemia [10]. It is well known that ARs are ubiquitously distributed in our body included the cells on the central nervous system (CNS) and on the peripheral blood cells [3]. Interestingly, ARs reproduce in the periphery a similar dysfunction present in the brain suggesting that the receptor alteration, easily measurable, could underlie disease mechanisms [11,12].

Previous studies have shown that A_1_ARs activation hampers Ca^2+^ influx inducing presynaptic inhibition and reducing excitatory neurotransmitters release and increases the conductance of K^+^ and Cl^-^ ions, decreasing neuronal excitability [13]. Thus, A_1_AR agonists ameliorate ischemic or excitotoxic neuronal damage after ischemia, even if the use of selective A_1_AR agonists is limited by the bad side effects such as sedation, bradycardia, and hypotension [14]. 

Many published papers suggest that A_2A_ARs have a dual role in cerebral ischemia: while A_2A_AR antagonists control excitotoxicity providing early protection, A_2A_AR agonists could give protracted protection by controlling leukocyte infiltration in the hours and days after ischemia [15]. Accordingly, A_2A_AR agonist/antagonist administration should be carefully evaluated in the function of the time after ischemia [16]. A great body of literature suggests that peripheral effects on A_2A_ARs located on blood cells are responsible for the protective effects of adenosine A_2A_AR agonists after ischemia. A_2A_ARs are expressed on cells of innate immunity such as macrophages, mast cells, monocytes, dendritic cells, neutrophils and microglia as well as on adaptive immunity cells such as lymphocytes [17]. It has been reported that, after focal ischemia induced by permanent middle cerebral artery occlusion, an upregulation of A_2A_ARs was present at the central level on neurons and microglia of ischemic striatum and cortex [18]. A_2A_AR subtype is localized not only at the central level but also peripherally on blood cells where reduces adhesion cell factor production, platelet aggregation and neutrophil activation, exerting, therefore, an antithrombotic, antioxidant and anti-inflammatory effect [7,19,20]. 

In CNS, A_2B_ARs are present in different cells and tissues including the hippocampus where they cause an increase of intracellular cAMP and the energy supply of neurons [21]. It is known that A_2B_ARs can be activated during hypoxic conditions when adenosine reaches pathological concentrations and their action may be cell-type dependent mediating both pro-inflammatory or anti-inflammatory effects [22].

Data in the literature about A_3_ARs role in the pathophysiology of cerebral ischemia are rather contradictory playing an initial protective role with A_1_ARs by inhibiting excitatory synaptic transmission [23]. However, A_3_AR prolonged activation increases excitotoxicity and the risk of damage, possibly via the activation of PKC and subsequent calcium increase suggesting that the protective or deleterious role of A_3_ARs depends on the severity and duration of ischemia [7].

It is well-known that following ischemic insult ATP is released, by apoptotic and necrotic cells, into the extracellular space and rapidly converted to ADP, AMP, and adenosine by the membrane-associated ecto-nucleotidases CD39 and CD73. Previous studies highlighted that transcellular metabolism, by CD39 and CD73, of ATP and ADP released by activated platelets can mitigate the amplification of the thrombotic nidus formation, resulting in the reduction of ischemic stroke damage [24]. The last product of CD73 activity is adenosine which, in the extracellular space represents a key modulator of mucosal and endothelial homeostasis such as mucosal integrity, vascular flow and leukocyte traffic [25]. In particular, adenosine controls vascular permeability and has immunosuppressive effects mainly through the activation of A_2A_ARs on immune cells. Hypoxia influences the adenosine signaling partly through hypoxia-inducible factor-1α (HIF-1α) stabilization, leading to the activation of ARs and CD73 [26]. Many published reports indicate that different pro-inflammatory cytokines such as IL-6 and IL-12, interferon beta and gamma, transforming growth factor beta (TGF-β) and adenosine itself regulate in a paracrine manner CD73 activity [27,28,29]. The importance of CD73 in cell-mediated immunity has been discovered in studies by using CD73 deficient mice and models of autoimmune uveitis [30]. In the literature, many studies have reported CD73 as a novel therapeutic target in cancer, multiple sclerosis, and chronic Toxoplasma gondii infection [31].

According to these findings and to the important role of adenosine and its receptors in different physiological and/or pathological conditions, we hypothesized an altered expression ARs and ectonucleotidases CD39 and CD73 in lymphocytes from patients affected by an ischemic stroke. To reach this purpose we analyzed in lymphocytes from patients’ blood samples the mRNA expression and receptor density and affinity of ARs, by real-time RT-PCR and by saturation binding assays, respectively. Moreover, the expression and frequency of ecto-nucleotidases CD39 and CD73 were evaluated by flow cytometry in total lymphocytes as well as in CD4^+^ and CD8^+^ T cell subsets. 

## 2. Materials and Methods

### 2.1. Ischemic Stroke Patients and Healthy Subjects

All patients enrolled in the study were recruited from the Neurology Unit of S. Anna Hospital, University of Ferrara, Italy. A total of 71 ischemic stroke patients and 70 control subjects were included. The demographic and clinical features of the subjects are listed in Table 1. Patients were classified on the basis of the ischemic insult, according to OCSP classification (Oxfordshire Community Stroke Project). This classification recognizes 4 different types of ischemic stroke named as follows: TACI (total anterior circulation infarcts), PACI (partial anterior circulation infarcts), LACI (lacunar circulation infarcts) and POCI (posterior circulation infarcts). The severity of the stroke consequences was assessed according to NIHSS (National Institutes of Health Stroke Scale or NIH Stroke Scale), a common and reliable method by which healthcare providers could objectively quantify the impairment caused by a stroke. The scores are: from 1 to 5 is slight impairment, 6-14 moderate impairment, 15–25 severe impairment, >25 very severe impairment. Patients were also categorized by subtypes of ischemic stroke mainly based on etiology determined following TOAST (Trial of Org 10172 in Acute Stroke Treatment) classification, which denotes five subtypes of ischemic stroke: LAA (large-artery atherosclerosis), CE (cardioembolism), SVA (small-vessel occlusion), Other (stroke of other determined etiology), and undetermined (stroke of undetermined etiology). Healthy control subjects (n = 70) were volunteers from Ferrara University Hospital Blood Bank. The study was approved by the local Ethics Committee of the University Hospital of Ferrara (Italy) (ethical code number: 170480; date of approval: 19 July 2017) and, in accordance with the principles outlined in the Declaration of Helsinki (informed consent was obtained from each participant).

### 2.2. Sample Collection and Human Lymphocyte Preparation

Peripheral blood of control individuals and ischemic stroke patients was used to obtain lymphocytes no later than 3–4 h after the samples were drawn. At the onset of each experiment, the human blood was placed to 6% by weight in the Dextran T500 solution (Sigma-Aldrich, St. Luis, MO, USA) for 60 min for the erythrocyte sedimentation. Leukocytes were centrifuged for 15 min at 100 × g and placed in distilled water at 4 °C to lyse the remaining erythrocytes. After the centrifugation for 5 min at 250 × g human lymphocytes were suspended in Krebs–Ringer phosphate buffer and stratified in 10 mL of Fycoll-Hypaque (GE Healthcare, Marlborough, MA, USA). After centrifugation, mononuclear cells were washed in 0.02 M phosphate-buffered saline at pH 7.2 containing 5 mM MgCl_2_ and 0.15 mM CaCl_2_. Then, the leukocytes were decanted and placed in a humidified incubator at 5% CO_2_ for 2 h at 37 °C to remove monocytes adhering to the culture flasks. The final suspension was a purified lymphocyte preparation containing at least 99% small lymphocytes and well-identified by morphological criteria. The lymphocytes were centrifuged in a hypotonic buffer at 20,000× *g* for 10 min to obtain membrane suspensions. After the centrifugation, the pellet was suspended in Tris-HCl 50 mM buffer pH 7.4 containing 2 UI/mL adenosine deaminase (Sigma-Aldrich) and incubated for 30 min at 37 °C. At the end of the incubation, the suspension was centrifuged at 40,000 × g for 10 min and the final pellet was used for radioligand binding assays. 

### 2.3. Real-Time Quantitative Polymerase Chain Reaction Assays

The acid guanidinium thiocyanate phenol method was used to extract total cytoplasmic RNA from lymphocyte of ischemic stroke patients and healthy subjects. Gene-specific fluorescently labeled TaqMan MGB probe (minor groove binder) was used in an ABI Prism 7700 Sequence Detection System (Applied Biosystems, Foster City, CA, USA). The Assays-on-Demand TM Gene Expression Products NM 000674, NM 000675, NM 000676 and NM 000677 were used for the RT-PCR of A_1_, A_2A_, A_2B_ and A_3_ARs, respectively. The endogenous control human β-actin kit was utilized for the RT-PCR of the reference gene, and the probe was fluorescent-labeled with VIC^TM^ dye (Applied Biosystems) [12].

### 2.4. Saturation Binding Assays to A_1_, A_2A_, A_2B_, and A_3_ARs

In A_1_AR saturation binding experiments, human lymphocyte membranes (60 μg of protein/assay) were incubated with 8 to 10 different concentrations of radioligand in the range 0.01–20 nM of [^3^H]-DPCPX ([^3^H]-1,3-dipropyl-8-cyclopentyl-xanthine, specific activity 120 Ci/mmol, Perkin Elmer, Boston, MA, USA). Non-specific binding was determined in the presence of 1μM DPCPX for an incubation time of 90 min at 25 °C.

The membrane suspension containing 60 μg of protein/assay was used to perform saturation binding experiments to A_2A_ARs and incubated for 60 min at 4 °C. The antagonist radioligand [^3^H]-ZM 241385 ([^3^H]-4-(2-[7-amino-2-(2-furyl)[1,2,4]-triazolo[2,3-a][1,3,5]triazin-5-ylamino]ethyl)phenol, specific activity 27 Ci/mmol, Biotrend, Köln, Germany) were used at various concentrations from 0.01 to 20 nM and non-specific binding was determined in the presence of ZM 241385 at the final concentration of 1μM.

Binding experiments with A_2B_AR antagonist radioligand in human lymphocyte membranes were performed using [^3^H]-MRE 2029F20 ([^3^H]-N-benzo[1,3]dioxol-5-yl-2-[5-(2,6-dioxo-1,3-dipropyl-2,3,6,7-tetrahydro-1H-purin-8-yl)-1-methyl-1H-pyrazol-3-yl-oxy]-acetamide, specific activity 123 Ci/mmol, GE Healthcare) and 1 μM MRE 2029F20 to determine the non-specific binding. Cell membranes at a concentration of 80 μg of protein/assay and [^3^H]-MRE 2029F20 from 0.01 to 30 nM were incubated for 60 min at 4 °C. 

To investigate the affinity and density of A_3_ARs, saturation binding experiments were assessed using [^3^H]-MRE 3008F20 ([^3^H]-5N-(4-methoxyphenylcarbamoyl)amino-8-propyl-2-(2-furyl)pyrazolo[4,3-e]-1,2,4-triazolo[1,5-c]-pyrimidine, specific activity 67 Ci/mmol, GE Healthcare) as radioligand and MRE 3008F20 1 μM was used to evaluate non-specific binding. The suspension membranes (80 μg of protein/assay) with [^3^H]-MRE 3008F20 (0.01–30 nM) were incubated at 4 °C for 150 min. 

Bound and free radioactivity were separated by filtering the assay mixture on Whatman GF/B glass fiber filters (Sigma-Aldrich) in a Brandel cell harvester (Brandel Inc., Gaithersburg, MD, USA). The filter-bound radioactivity was measured in a 2810 TR liquid scintillation counter (Perkin Elmer) [12].

### 2.5. Flow Cytometry Analysis

Whole blood samples were added with Red Blood Cells Lysis Buffer containing NH_4_Cl 150 mM, NaHCO_3_ 10mM, EDTA 1mM for 30 min, room temperature (RT), in order to lyse red blood cells. The cell suspension was centrifuge 350× *g* for 5 min. The supernatant was aspirated and the pellet resuspended in Staining Buffer (PBS with 5% FBS and 0.1% NaN_3_). Samples staining with directly conjugated antibodies for CD4 (PE-Cy5.5, clone SK3, eBioscience, San Diego, CA, USA), CD8 (PE-Cy7, clone SK1, eBioscience), CD39 (Alexa Fluor 488, clone A1, eBioscience) and CD73 (R-PE, clone AD2, eBioscience) was performed on 100 μl of each sample for 15 minutes, RT. After antibody incubation samples were washed with Staining buffer and analyzed. For compensation, single stained controls were used. Flow cytometry analysis was performed on Attune NxT cytometer (Thermo Fisher Scientific, Paisley, UK) using negative and fluorescence-minus-one (FMO) controls, data analysis was carried out using Attune NxT software version 3.1.2 (Thermo Fisher Scientific). Flow cytometry data for ectonucleotidases are reported both as percentages of CD39 or CD73 positive lymphocytes and Median Fluorescence Intensity (MFI) values. Percentage represents the frequency of CD39 or CD73 expressing cells among total lymphocytes and specific CD4+ and CD8+ subsets. MFI values represent the expression levels of CD39 or CD73 on lymphocyte cell surface.

### 2.6. Statistical Analysis

The protein concentration was determined by the Bio-Rad method by using, as a reference standard, bovine albumin [12]. Saturation binding parameters such as affinity, expressed as K_D_ values, and the receptor densities identified as Bmax values were calculated for a system of one- or two-binding site populations by means of a non-linear curve fitting using GraphPad Prism software version 6.0 (GraphPad Software). Data are reported as mean ± SEM of different independent experiments as indicated in the legend of the figures or in the text. Analysis of data was subsequently performed by one-way analysis of variance (ANOVA) and the differences between the groups were analyzed with Bonferroni’s test. A non-parametric two-tailed Mann–Whitney U-test was used for comparison between two groups, as for flow cytometry experiments. Values of *p* < 0.05 were considered significant

## 3. Results

### 3.1. A_2A_AR mRNA Expression is Up-Regulated in Lymphocytes of Ischemic Stroke Patients

**Figure 1:**
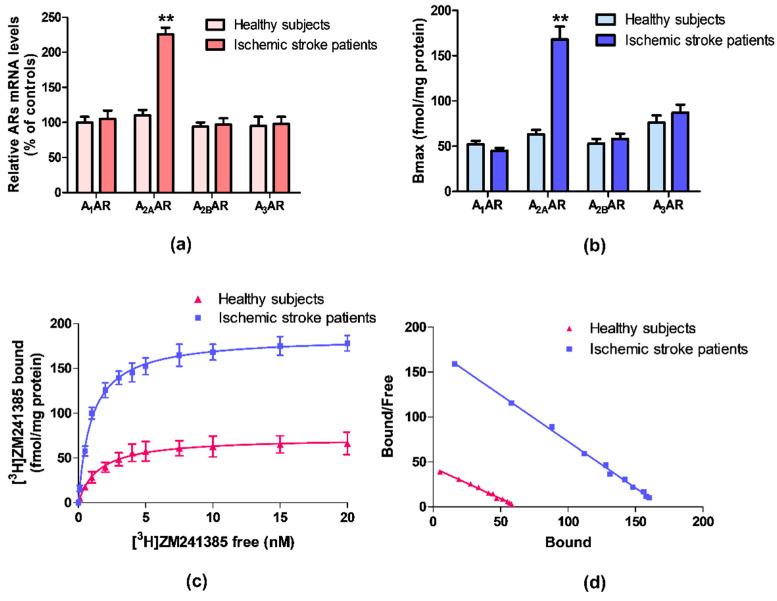
(**a**) Relative adenosine receptors (ARs) mRNA levels determined by reverse transcription (RT)-polymerase chain reaction (PCR) in human lymphocytes from ischemic stroke patients (n = 50) and control subjects (n = 50). (**b**) Density of A_1_, A_2A_, A_2B_, and A_3_ARs, expressed as Bmax, in lymphocytes derived from ischemic stroke patients (n = 50) in comparison to control subjects (n = 50). (**c**) Saturation curve and (**d**) Scatchard plot of [^3^H]-ZM 241385 to A_2A_ARs in lymphocyte membranes derived from ischemic stroke patients (n = 50) and control subjects (n = 50). Data are expressed as the mean ± SEM. ** *p* < 0.01 vs control group.

ARs mRNA expression was evaluated by quantitative RT-PCR assay in lymphocytes obtained from ischemic stroke patients and healthy subjects. Figure 1a reports the relative A_1_, A_2A_, A_2B_, and A_3_ARs mRNA levels determined by RT-PCR in human lymphocytes of healthy subjects and ischemic stroke patients. Among these receptors, only A_2A_ARs mRNA levels were significantly increased in patients in comparison to control subjects. No differences were found in the mRNA expression of A_1_, A_2B,_ and A_3_ARs. 

### 3.2. A_2A_AR Increase in Density and Affinity in Lymphocytes of Ischemic Stroke Patients

Saturation binding experiments allowed to determine the density, expressed as Bmax, and the affinity, expressed as K_D_, of ARs in ischemic stroke patients’ lymphocytes in comparison to healthy subjects. Figure 1b highlights a significant increase in A_2A_ARs density in lymphocytes from ischemic stroke patients, while no differences in density values of the other ARs subtypes were found. Saturation curves and Scatchard plot of [^3^H]-ZM 241385 in human lymphocytes, confirmed the up-regulation of A_2A_ARs in ischemic stroke patients compared with healthy subjects (Figure 1c,d).

The affinity and density values of ARs in lymphocyte membranes of ischemic stroke patients and healthy subjects are reported in Table 2. The affinity of the radioligand [^3^H]-ZM 241385 for A_2A_ARs is increased from 1.48 ± 0.10 to 0.97 ± 0.04 nM, in lymphocytes from patients compared with the control group. Interestingly, the A_2A_ARs density was significantly increased in patients reaching a 2.7-fold increment from 63 ± 5 in healthy subjects to 168 ± 14 fmol/mg protein. No differences in density and affinity values were detected for other ARs subtypes.

### 3.3. CD4 and CD8 Expression on Lymphocytes of Ischemic Stroke Patients

**Figure 2:**
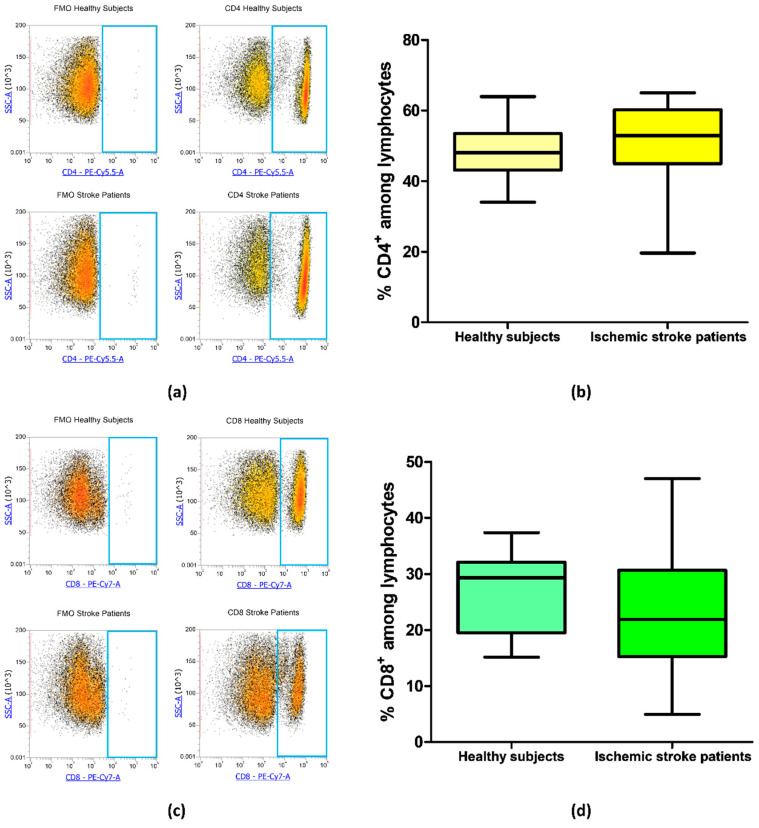
(**a**) Exemplary flow cytometry density plots showing Fluorescence Minus One (FMO) control and CD4 stained cells in lymphocytes from healthy subjects and ischemic stroke patients. (**b**) Box and whiskers plot showing the percentage of CD4^+^ lymphocytes in healthy subjects (n = 20) and ischemic stroke patients (n = 21)**.** (**c**) Representative density plots showing FMO control and CD8 stained cells in lymphocytes from healthy subjects and ischemic stroke patients. (**d**) Graphical representation of flow cytometry data showing the percentage of CD8^+^ lymphocytes in healthy subjects (n = 20) and ischemic stroke patients (n = 21)**.** Data are shown as the median, interquartile range and the minimum and maximum values.

Blood samples from 21 ischemic stroke patients and 20 healthy subjects were analyzed by flow cytometry to evaluate the frequency of CD4^+^ and CD8^+^ T cells in order to identify if there were differences in CD4/CD8 ratio in patients in comparison to healthy subjects. The frequency of CD4^+^ was equal to 48.45% ± 1.65% and 51.32% ± 2.30% in controls and ischemic stroke patients, respectively (*p* = 0.18, Figure 2a,b). Regarding CD8^+^ lymphocytes, ischemic stroke patients presented a mean percentage of 22.76% ± 2.21% and controls a mean value of 27.00% ± 1.56% (*p* = 0.09, Figure 2c,d). These data suggest that there were no significant differences in the percentage of CD4^+^ and CD8^+^ lymphocytic subpopulation in ischemic stroke patients in comparison to control subjects.

### 3.4. CD73 and CD39 Expression on Lymphocytes of Ischemic Stroke Patients

**Figure 3:**
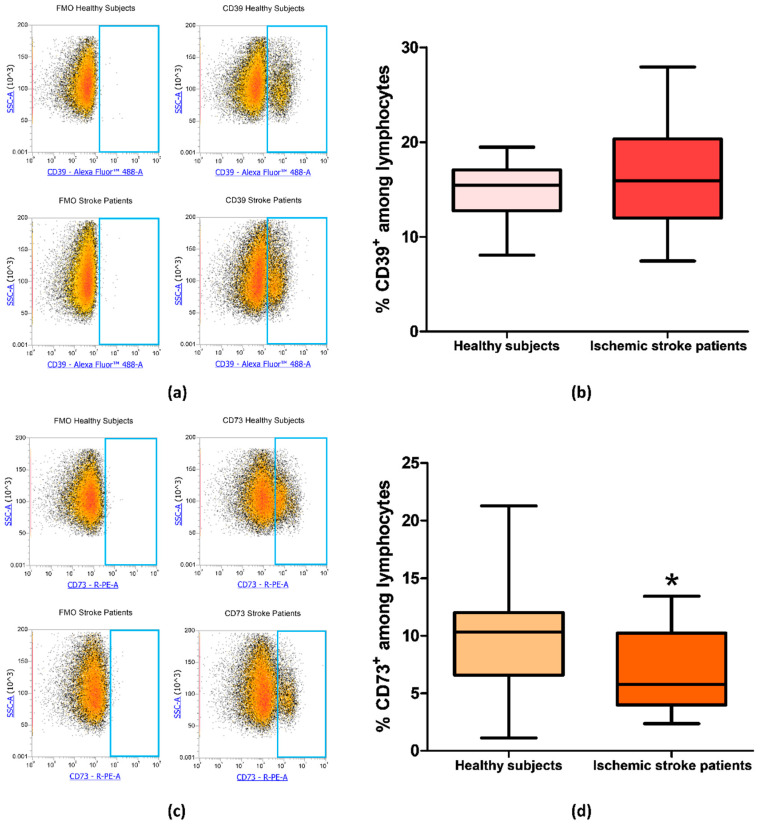
(**a**) Representative flow cytometry density plots showing FMO control and CD39 stained cells in lymphocytes from healthy subjects and ischemic stroke patients. (**b**) Graphical representation of flow cytometry data showing the percentage of CD39^+^ lymphocytes in healthy subjects (n = 20) and ischemic stroke patients (n = 21)**.** (**c**) Representative density plots showing FMO control CD73 stained cells in lymphocytes from healthy subjects and ischemic stroke patients. (**d**) Box and whiskers plot showing the percentage of CD73^+^ lymphocytes in healthy subjects (n = 20) and ischemic stroke patients (n = 21)**.** Data are shown as the median, interquartile range and the minimum and maximum values. * *p* < 0.05 vs healthy subjects.

Since CD73 and CD39 are important enzymes in adenosine metabolism, their expression was investigated by flow cytometry on peripheral blood lymphocytes of ischemic stroke patients (n  =  21) and healthy subjects (n  =  20). The percentage of CD39^+^ and CD73^+^ lymphocytes are reported in Table 3. In particular, the proportion of CD39^+^ lymphocyte in control subjects compared to patients did not show a significant difference with frequency values of 14.82% ± 0.66% and 16.22% ± 1.16%, respectively (*p* = 0.49, Figure 3a,b). Interestingly, a statistically significant decrease in CD73^+^ population in patients’ lymphocytes was found, with a lower frequency in patients (6.98% ± 0.77%, p = 0.0393) than in controls (10.04% ± 1.10%, Figure 3c,d). Frequency values for CD39 and CD73 positive lymphocytes are summarized in Table 3.

**Figure 4:**
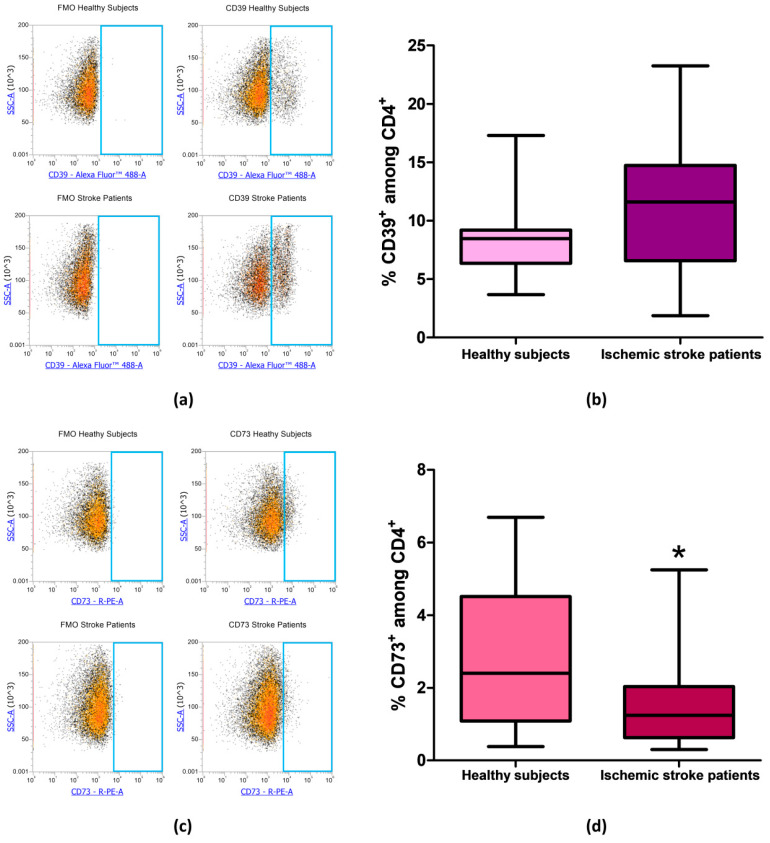
(**a**) Representative flow cytometry density plots showing FMO control and CD39 stained cells in CD4^+^ lymphocytes from healthy subjects and ischemic stroke patients. (**b**) Graphical representation of flow cytometry data showing the CD39^+^ percentage of CD4^+^ lymphocytes in healthy subjects (n = 20) and ischemic stroke patients (n = 21)**.** (**c**) Exemplary density plots showing FMO control and CD73 stained cells in CD4^+^ lymphocytes from healthy subjects and ischemic stroke patients. (**d**) Box and Whiskers plot showing the proportion of CD73^+^cells among CD4^+^ lymphocytes in healthy subjects (n = 20) and ischemic stroke patients (n = 21)**.** Data are shown as the median, interquartile range and the minimum and maximum values. * *p* < 0.05 vs healthy subjects.

After evaluating the expression of CD39 and CD73 in total lymphocytes we focused on CD4**^+^** and CD8**^+^** lymphocytes subpopulation, in order to determine whether the reduction in CD73 percentage in patients’ total lymphocytes was confirmed also in specific T cell subset. Even in CD4^+^ lymphocytes, we found no differences in the percentage of CD39^+^ cells between patients and controls. Percentage of CD39^+^ among CD4^+^ lymphocytes was equal to 8.56% ± 0.74% and to 11.35% ± 1.29% in healthy controls and ischemic stroke patients, respectively (*p* = 0.13, Figure 4a,b, Table 3). The difference in the frequency of CD73^+^ cells between healthy subjects and ischemic stroke patients was significant, within the CD4^+^ population, with values of 2.96% ± 0.45% and 1.47% ± 0.25%, respectively (*p* = 0.0315, Figure 4c,d, Table 3). 

**Figure 5:**
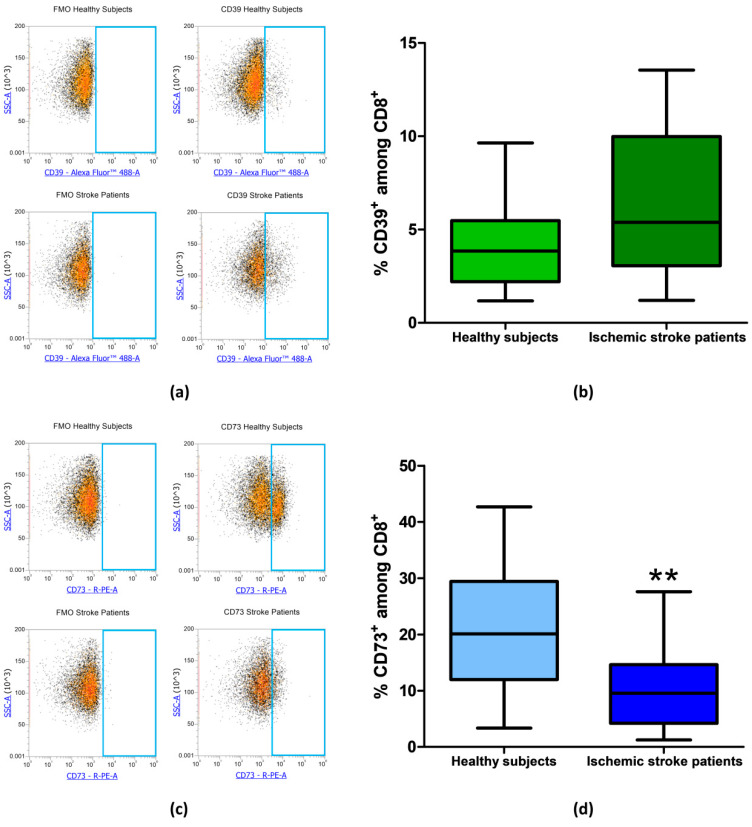
(**a**) Exemplary flow cytometry density plots showing FMO control and CD39 stained cells in CD8^+^ lymphocytes from healthy subjects and ischemic stroke patients. (**b**) Graphical representation of flow cytometry data showing the CD39^+^ percentage of CD8^+^ lymphocytes in healthy subjects (n = 20) and ischemic stroke patients (n = 21)**.** (**c**) Representative density plots showing FMO control and CD73 stained cells in CD8^+^ lymphocytes from healthy subjects and ischemic stroke patients. (**d**) Box and Whiskers plot showing the proportion of CD73^+^cells among CD8^+^ lymphocytes in healthy subjects (n = 20) and ischemic stroke patients (n = 21)**.** Data are shown as the median, interquartile range and the minimum and maximum values. ** *p* < 0.01 vs healthy subjects.

The frequency of CD39^+^ and CD73^+^ among CD8^+^ lymphocytes was then measured in healthy subjects and ischemic stroke patients. In CD8^+^ T-cell subset, CD39^+^ cells did not significantly differ between healthy controls and ischemic stroke patients with frequency values of 4.09% ± 0.53% and 6.50% ± 0.90%, respectively (*p* = 0.0565, Figure 5a,b, Table 3). Notably, with respect to CD4^+^, in CD8^+^ lymphocytes, even a more significant reduction of CD73^+^ population was observed with a value of 10.54% ± 1.48% in comparison to healthy subjects were a frequency of 21.04% ± 2.54% was found (*p* = 0.002, Figure 5c,d, Table 3). 

To investigate whether in addition to the frequency alteration of CD73 positive lymphocytes there was also a variation of the expression levels of CD39 or CD73 on the lymphocyte cell surface, Median Fluorescence Intensity values were calculated. Table 4 reports the Median Fluorescence Intensity (MFI) values indicating CD39 and CD73 expression in total, CD4^+^ and CD8^+^ lymphocytes from ischemic stroke patients in comparison to healthy controls. It is interesting to notice that only in CD8^+^ lymphocytes there is a significant reduction of CD73 expression in patients in comparison to control subjects with MFI = 4239 ± 184 and MFI = 5197 ± 246 respectively (*p* = 0.0075). No significant differences regarding CD39 and CD73 expression were found in total lymphocytes and in CD4^+^ subpopulation between healthy subjects and ischemic stroke patients.

## 4. Discussion

Ischemic stroke is one of the leading causes of morbidity and mortality affecting millions of people worldwide [32]. Cell injury represents an inevitable consequence of cerebral ischemia and the related cell death pathways are well-reported and primarily due to the increase of glutamate [6]. Increasing evidence demonstrates that adenosine and ARs under pathological conditions are associated with either the induction of cell death and cerebral damage or neuroprotective effect [7]. It is well established that ARs expressed in human peripheral cells could represent a similar dysfunction in the brain suggesting a possible relevant mechanism in the disease. As a consequence, a measurable significant change in biological processes can be well quantified in accessible blood cells [11,12,33].

In this paper, the presence of ARs in lymphocytes from ischemic stroke patients compared to healthy subjects was investigated. Noteworthy, only A_2A_ARs were up-regulated in patients while no differences were found for A_1_, A_2B_ or A_3_ARs. In particular, A_2A_ARs alteration has been found in receptor binding parameters, density, and affinity and also confirmed at mRNA level. Increasing evidence shows that A_2A_ARs can form homo-oligomers and hetero-oligomers with other G protein-coupled receptors. The increase of [^3^H]-ZM 241385 affinity for A_2A_ARs observed in lymphocytes from ischemic stroke patients could suggest a different proportion of homo-oligomers and hetero-oligomers following the upregulation A_2A_ARs. A similar alteration of the binding parameters evaluated in human blood cells well correlated with clinical phenotype in neurodegenerative diseases, such as Parkinson’s disease, in which A_2A_AR antagonists have shown a therapeutic effect both in experimental models and in clinical trials [12,34]. Moreover, it has been shown that A_2A_ARs are implicated in other neurodegenerative pathologies such as Amyotrophic Lateral Sclerosis and Huntington’s disease where A_2A_AR alteration correlated with disease stage and genetic parameters [3,11,35]. 

Until now, there are limited articles in the literature on the presence of ARs in lymphocytes from ischemic stroke patients and the most relevant results available are on the effect of adenosine and ARs in hypoxic/ischemic conditions [2,15,16]. In particular, different studies suggest that A_2A_ARs have a dual role in cerebral ischemia: A_2A_AR antagonists provide early protection via centrally mediated control of excessive excitotoxicity, while A_2A_AR agonists provide protracted protection by controlling massive blood cell infiltration in the hours and days after ischemia [16]. Accordingly, A_2A_AR agonists and/or antagonists should be carefully evaluated in function of the time after stroke by using doses that do not modify blood pressure and heart rate [14]. In the CNS, they are involved in neuroprotection against brain ischemia by increasing NGF and BDNF, important factors involved in the recovery of brain activities after an ischemic insult [36]. A_2A_ARs are present both on cells of innate immunity and on lymphocytes, where they have a prominent role in the suppression of inflammatory responses [17]. In many previous works, we investigated the effect of cultured A_2A_AR stimulation in lymphocytes from chronic inflammatory and neurodegenerative disease patients and healthy subjects. In particular, the A_2A_AR agonist CGS21680 inhibited NF-κB activation, reduced inflammatory cytokines release and metalloproteinases production [37,38,39,40]. A great body of literature suggests that peripheral effects on A_2A_ARs located on blood cells are responsible for the protective effects of A_2A_AR agonists after ischemia-reperfusion injury by inhibiting inflammatory processes [17,41]. As a consequence A_2A_AR agonists could be relevant in a wide therapeutic time-window of hours and even days after stroke considering their important role in inflammatory responses acting as anti-inflammatory agents [14]. 

Given the complex nature of the adenosinergic system, we focused our attention on the analysis of surface molecules involved in adenosine generation such as CD39 and CD73. In our experimental conditions, flow cytometry analysis reveals a significant reduction of CD73 in total lymphocytes from ischemic stroke patients in comparison to control subjects, findings that were also confirmed in CD4^+^ and CD8^+^T-cell subsets. Different lines of evidence demonstrated that adenosine, produced by the coordinated function of CD39 and CD73, has a pivotal role in protecting tissue against hypoxic and ischemic insults. Many studies, using genetic knockout or pharmacological inhibition of CD39 and CD73, highlighted that these enzymes counteract vascular permeability and leukocyte infiltration during local hypoxia [26,42,43]. The lack of protection from inflammation observed in CD39 and CD73 knockout mice could be reversed through ARs stimulation by exogenous administration of NECA, a non-selective AR agonist, or by exogenous reconstitution with soluble forms of CD39 and CD73 [24]. Other studies in the literature report that both CD4^+^ and CD8^+^ T cell numbers were increased in the ischemic brains of CD73 knockout mice [44]. In the present study, no differences were found on the proportion of CD4^+^ and CD8^+^ lymphocytes in ischemic stroke patients in comparison with healthy subjects. In a rat model of stroke has been observed that there is an increase of CD73 expression in infarcted tissue and hypoxia drives a transcriptional increase of both surface ecto-nucleotidases, CD39 and CD73 [45,46]. Hypoxia can also induce the expression of CD73 via binding of hypoxia-inducible factor (HIF)1-α to the CD73 promoter region [26,42]. The hypoxia-mediated CD73 upregulation suggests that the decreased frequency of CD73 observed in our study in lymphocytes from ischemic stroke patients, may not represent a consequence but rather a constitutive factor prior to the ischemic stroke. Adenosine levels increase substantially in hypoxic conditions and the accumulation of adenosine is largely due to the activity of surface-expressed CD73. In addition, it is well-recognized that decreased expression of CD73 results in reduced adenosine production [47]. However, the reduced frequency of CD73 positive lymphocytes is probably not a sufficient condition for the reduction of adenosine plasma levels. In fact, in a study conducted on stroke patients, adenosine plasma levels were found to be slightly above the normal range upon admission, peaked on day 3 and then decreased towards the normal range [48]. However, it is should be emphasized that adenosine plasma levels are regulated by many cells type and different distinct mechanisms [2]. An interesting consequence of reduced CD73 activity has been reported in a study performed in mice lacking CD73. CD73^−/−^ mice subjected to photothrombotic arterial occlusion exhibited a larger cerebral infarct volume and more tissue leukosequestration than wild type mice [24]. This suggests a neuroprotective effect of CD73 in cerebral ischemia as a modulator of brain inflammation and immune function.

## 5. Conclusions

In conclusion, this paper reports, for the first time, the presence of an A_2A_ARs up-regulation in lymphocytes obtained from ischemic stroke patients when compared with healthy subjects. In particular, RT-PCR and saturation binding experiments revealed that A_2A_ARs expression, density, and affinity were significantly increased in patients’ lymphocytes. Given the anti-inflammatory action of A_2A_AR activation by adenosine, this alteration could represent a compensatory mechanism to counteract excessive inflammation and leukocyte infiltration following brain ischemia. On the other hand, in lymphocytes from ischemic stroke patients, we observed a significant decrease in the frequency of the adenosine forming enzyme ecto-5’-nucleotidase CD73. Evaluating the specific T-cell subset, a more significant reduction of CD73 positive population was found in CD8^+^ T-lymphocytes. The decreased possibility of adenosine generation following the reduction of CD73 is likely to cause an enhanced inflammatory response, despite the compensatory A_2A_AR upregulation observed in patients’ lymphocytes. These observations corroborate the notion already reported in different papers in the literature on the potential therapeutic use of selective A_2A_AR agonists to counteract post-ischemic inflammation and leukocyte infiltration in stroke patients. Further studies are needed to investigate if the decreased frequency of CD73 in lymphocytes, and in particular in CD8^+^ T-lymphocytes could represent, in combination with A_2A_ARs upregulation, a blood biomarker of ischemic stroke. Furthermore, these data could pave the way for the development of specific and more targeted therapeutic approaches, exploiting the complex and prominent role of the adenosinergic system in ischemic stroke.

## Figures and Tables

**Table 1:** Demographic and clinical features of ischemic stroke patients and healthy subjects enrolled in the study.

**Ischemic Stroke Patients**	**n = 71**
Demographic features	
N° female/male	35/36
Age	78.31 ± 13.13
Hypertension	48
Diabetes	12
Smoke	10
Atrial fibrillation	26
OCSP patients classification	
TACI	19
PACI	36
POCI	8
LACI	8
NIHSS	
Slight impairment (1–5)	26
Moderate impairment (6–14)	26
Severe impairment (15–25)	19
Very severe impairment (>25)	0
TOAST Classification	
LAA	20
CE	34
SVA	7
Other	1
Undetermined	9
**Healthy Subjects**	**n = 70**
Demographic features	
N° female/male	31/39
Age	61.24 ± 12.17

OCSP classification: Oxfordshire Community Stroke Project classification; TACI: total anterior circulation infarcts; PACI: partial anterior circulation infarcts; LACI lacunar circulation infarcts; POCI: posterior circulation infarcts; NIHSS: National Institutes of Health Stroke Scale or NIH Stroke Scale; TOAST classification: Trial of Org 10172 in Acute Stroke Treatment classification; LAA: large-artery atherosclerosis; CE: cardioembolism; SVA: small-vessel occlusion.

**Table 2:** Adenosine receptors binding parameters in lymphocytes from patients with ischemic stroke in comparison with healthy subjects. Data are expressed as the mean ± SEM. Differences were considered significant at a value of ** *p* < 0.01 vs healthy controls.

	A_1_ARs	A_2A_ARs	A_2B_ARs	A_3_ARs
Healthy subjects (n = 50)				
K_D_ (nM)	1.74 ± 0.11	1.48 ± 0.10	2.11 ± 0.20	1.87 ± 0.14
Bmax (fmol/mg protein)	52 ± 4	63 ± 5	53 ± 5	76 ± 8
Ischemic stroke patients (n = 50)				
K_D_ (nM)	1.58 ± 0.12	0.97 ± 0.04**	2.34 ± 0.16	1.91 ± 0.11
Bmax (fmol/mg protein)	45 ± 3	168 ± 14**	58 ± 6	87 ± 9

**Table 3:** Sizes of different lymphocytic populations expressing CD39 and CD73 from healthy subjects (n = 20) and ischemic stroke patients (n = 21). Population size is given in the mean percentage ± SEM. Non-parametric two-tailed Mann–Whitney U-test was used to test for significance. * *p* <0.05; ** *p* < 0.01.

	CD39 (%)		CD73 (%)	
	Healthy Subjects	Ischemic Stroke Patients	Healthy Subjects	Ischemic Stroke Patients
All lymphocytes	14.82 ± 0.66	16.22 ± 1.16	10.04 ± 1.10	6.98 ± 0.77*
CD4+ lymphocytes	8.56 ± 0.74	11.35 ± 1.29	2.96 ± 0.45	1.47 ± 1.10*
CD8+ lymphocytes	4.09 ± 0.53	6.50 ± 0.90	21.04 ± 2.54	10.54 ± 1.48**

**Table 4:** Expression level of both CD39 and CD73 ectonucleotidases in lymphocytes from healthy subjects (n = 20) and ischemic stroke patients (n = 21). Data are reported as median fluorescence intensity (MFI) ± SEM. Non-parametric two-tailed Mann–Whitney U test was used to test for significance; ** *p* < 0.01.

	CD39 (MFI)		CD73 (MFI)	
	Healthy Subjects	Ischemic Stroke Patients	Healthy Subjects	Ischemic Stroke Patients
All lymphocytes	5389 ± 515	4502 ± 416	10084 ± 588	11436 ± 986
CD4+ lymphocytes	4783 ± 258	4715 ± 380	6983 ± 234	7058 ± 396
CD8+ lymphocytes	2640 ± 208	2357 ± 212	5197 ± 246	4239 ± 184**
